# Spectroscopic methods for assessment of hand sanitizers

**DOI:** 10.1007/s11696-022-02208-x

**Published:** 2022-04-25

**Authors:** Soumyabrata Banik, Sindhoora Kaniyala Melanthota, Anjana Anandan Vannathan, Krishna Kishore Mahato, Sib Sankar Mal, Nirmal Mazumder

**Affiliations:** 1 Department of Biophysics, Manipal School of Life Sciences, Manipal Academy of Higher Education, Manipal, Karnataka 576104 India; 2 Materials and Catalysis Lab, Department of Chemistry, National Institute of Technology Karnataka, Surathkal, Karnataka 575025 India

**Keywords:** Disinfectant, Hand sanitizers, Fourier transform infrared spectroscopy, Raman spectroscopy

## Abstract

Sanitization of inanimate objects or body surfaces using disinfectant is essential for eliminating disease-causing pathogens and maintaining personal hygiene. With the advent of health emergencies, the importance and high demand for hand sanitizers (HS) are observed in everyday life. It is also important to know the constituent added to formulate HS, as the presence of harsh chemicals can cause skin irritation. In this study, different spectroscopic techniques were used to assess several commercially available HS along with the in-house prepared HS as per the WHO protocol. Fourier transform infrared spectroscopy and Raman spectroscopy identified the different HS chemical bonds and quantified the amount of alcohol and water in the HS. Varying amount of alcohols in HS, calibration profile was generated to identify its amount in commercial samples. Further, the commercial samples were also checked for contaminants whose presence in the HS might bring down its sanitization efficacy.

**Supplementary Information:**

The online version contains supplementary material available at 10.1007/s11696-022-02208-x.

## Introduction

Disinfection is the process of eliminating microbes from inanimate objects. This process is considered less effective than sterilization, as the latter unitizes physical or chemical methods to destroy all microbial forms (Guideline for Disinfection and Sterilization in Healthcare Facilities(2018).https:, www.cdc.gov, infectioncontrol, guidelines, disinfection, introduction.html(accessed 2021–03-01). 2008). However, sterilization is time-consuming, expensive, and requires a dedicated setup. Alternatively, disinfectants have gained immense popularity because they have shown comparable efficacy with fast action. Disinfectants are of two types: chemicals-based, such as alcohol, aldehydes, and hydrogen peroxides, or non-chemical-based, like germicidal irradiation by ultraviolet rays. Alcohol-based hand rubs or hand sanitizers (HS) are commonly used disinfectants to maintain proper hand hygiene. HS are exogenous disinfectants and clinically proven to reduce the growth of several micro-organisms in hand. Since its invention, it has been extensively used by the public, including healthcare professionals, doctors, and even scientists due to its easy availability and effectiveness (Hilburn et al. 2003). Extensive use of HS has been observed with the emergence of public health crisis such as like pandemics or disease outbreaks. COVID-19 (Coronavirus Disease-2019) spreads through aerosol, and these droplets are often found on various surfaces. Studies have shown that by maintaining effective hand hygiene, the transmission of the virus can be reduced significantly (Kampf et al. 2020; Siddharta et al. 2017). The US Centers for Disease Control and Prevention recommends using HS as an effective way to avoid falling sick and prevent the spread of contagious diseases (Prevention of Coronavirus Disease 2019).

HS are either commercially available or can be prepared using the WHO-approved protocol (Guide to local production: WHO-recommended handrub formulations. 2010). Most of these HS contain 60% to 95% ethanol or isopropanol and other constituents like glycerol, hydrogen peroxide, distilled water, perfumes, and various additives whose concentrations often vary among the different brands. Alcohol denatures the microbial proteins byinterfering with their cellular metabolism or destroying the cell walls of microbe, thereby eliminating them (Jing et al. 2020). Therefore, the amount of alcohol in HS signifies its efficacy in the process of disinfection. Hydrogen peroxide acts to inactivate the contaminating spores that come in contact with hands and surfaces, whereas glycerol, a humectant, maintains skin moisture. Apart from these constituents, the manufacturers include some additional components such as essential oils, vitamins, or perfumes, which bring a soothing effect to the skin. Thickening ingredients are also added to increase the viscosity of the HS, and the chemical flavoring compounds improve the product's aesthetic. These additional chemical agents can cause severe local irritations and other skin implications (Jing et al. 2020). Studies suggest that many companies utilize harsh chemicals such as triclosan or benzene, which can trigger allergic responses (Bissett 2007).

With the rising demand for HS, differences in its composition are likely to emerge, with most manufacturers failing to adhere to the specified guidelines for effective microbe elimination, resulting in a false sense of antisepsis (Shruthi et al. 2020). Therefore, qualitative and quantitative analysis of the HS to determine its constituents is fundamental and need of the hour. The present work proposes using electromagnetic radiation-based spectroscopic techniques as an alternative approach for quantitative and qualitative analysis of alcohol-based HS. Spectroscopy methods such as Fourier transform infrared (FTIR) and Raman were used complementary to each other to detect different functional groups and quantify their constituents of HS. Moreover, volatile compounds such as perfumes present in the HS are difficult to detect with FTIR spectroscopy, whereas UV–Visible absorbance and fluorescence are handier for such detections. Therefore, it is important to use different spectroscopic techniques to complement each other for the efficient assessment of multi-compound solutions like HS. Further, all the techniques discussed in this study are available in most research and quality control laboratories, they do not require extensive sample preparation, and are easy to use.

## Materials and methods

### Materials

Commercial HS from eight different brands (labeled as A-H), purchased from the local market areused in the experiments. The composition of the commercial HS, as mentioned by the manufacturer, is described in Table S1 (see supplementary information). Further, by following  the WHO-approved protocol (as mentioned in Table S2), HS is freshly prepared in-house with varying concentrations of alcohols (ethanol and isopropanol from 50 to 100%), glycerol, and 3% hydrogen peroxide. The samples were used for spectroscopic analysis at room temperature. The raw materials used for the in-house preparation of HS and as analytical standards include ethanol (CH_3_CH_2_OH) [99.99% V/V], glycerol (C_3_H_8_O_3_) [99.9% V/V], hydrogen peroxide (H_2_O_2_) [30%], methanol (CH_3_OH) [99.99% V/V], and isopropanol (CH_3_CH_2_ CH_2_OH) [99.99% V/V] were purchased from Merck Life Sciences Pvt. Ltd., India in addition to distilled water.

### Methods

Different spectroscopic techniques were used in this study to evaluate HS. ATR-FTIR spectra were recorded using FTIR Spectrometer (Alpha II, Bruker, USA). HS samples (~ 50 µL) were loaded into the ATR, and spectra were acquired in the range of 600–4000 cm^−1^ to analyze HS’s chemical composition. A portable near-IR Raman spectrometer AvaRaman-785 TEC (Avantes BV, The Netherlands) was used to record and analyze Raman spectra of various HS. The system is equipped with a diode laser (wavelength and bandwidth are 785 nm and < 0.2 nm, resolution 7 cm^−1^ respectively), spectrometer (AvaSpec-ULS2048LTEC, Avantes BV, The Netherlands) with grating (785–1080 nm), Raman probe, and sample holder. The samples were loaded (200 µL in each well) in a 96-well plate (Microplate 96/F, Eppendorf, Germany), and the laser was focused using a Raman probe, placed perpendicular to the plate at a focal length of ~ 1 cm for spectrum acquisition. A Raman spectrum was recorded from the blank wall for the background vibrational spectrum subtraction. The baseline correction, spectral smoothing, and spike removal from the Raman spectrum were performed with Spectragryph spectral analysis software (version 1.2.14).  Further, the FTIR and pre-processed Raman spectrum of HS samples were plotted using OriginPro software (version 2020) (OriginLab Corporation, USA) by considering intensity along Y-axis and wavenumber along X-axis.

UV–Vis absorbance and fluorescence spectroscopic measurements were acquired using a Varioskan Flash spectrophotometer (Thermo Scientific, USA). To record the spectrum, samples (200 µL in each well) were loaded in triplicates in a 96-well plate. The absorbance spectra were recorded using the SkanIt™ Software 2.4.3 RE software over the spectral range of 250–500 nm, having a scanning wavelength of step size 2 nm with the bandwidth being 5 nm and measurement time of 100 ms. For fluorescence signal measurement, 350 nm was used as the excitation wavelength. The emission was measured from 380 to 600 nm, with a step size of 2 nm. SkanIt™ Software 2.4.3 RE was used for the spectral acquisition with a measurement time of 100 ms. All the graphs were plotted and analyzed using OriginPro software (version 2020) (OriginLab Corporation, USA).

## Results and Discussion

### FTIR Spectroscopy

**Fig. 1:**
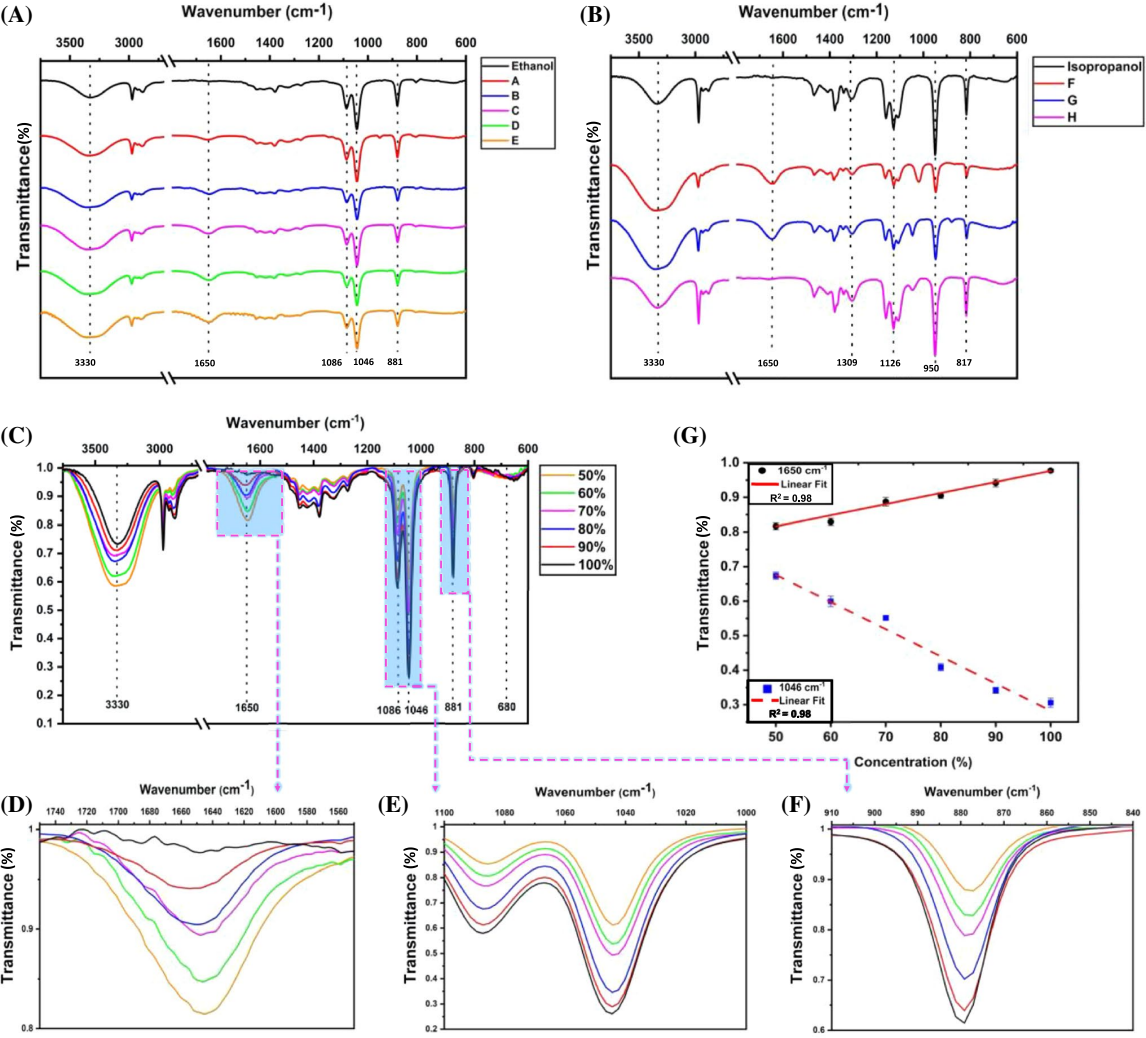
FTIR assessments of HS; **A**, **B** Ethanol and isopropanol-based commercial HS along with the spectra of ethanol and isopropanol. The major spectral features associated with alcohols and water are indicated. **C**–**G** FTIR analysis of in-house prepared HS. **C** Shows the spectra of in-house prepared HS with various concentrations of ethanol. **D**–**F** The variation of the FTIR transmittance intensity at 881 and 1046 cm^−1^ is a function of ethanol concentration, whereas at 1650 cm^−1^ is a function of water. **G** Integrated linear plots with varying alcohol and water concentration. The data are plotted as a function of transmittance intensity. The statistical values are estimated for the linear regression

FTIR is a type of vibrational spectroscopy that uses infrared radiation to record the absorbance or transmittance of the sample to analyze its chemical properties. In the present study, it was used to assess the commercially available HS and the different grades of HS prepared in-house according to the WHO protocol. Because it is recommended to use 70–95% ethanol or isopropanol in HS, it is critical to precisely measure the amount of alcohol. Figure 1 shows the FTIR spectra of various commercial HS and their corresponding assignment of different chemical bonds, which are summarized in Table 1. Upon spectral analysis of commercial HS, they were found to contain either ethanol or isopropanol and were divided into two groups: HS with ethanol (A, B, C, D, E) indicated as Fig. 1A and isopropanol (F, G, H) as represented in Fig. 1B. The FTIR spectra of ethanol and isopropanol are also illustrated in Fig. 1, which inferred that ethanol has a characteristic peak at 881 cm^−1^ and isopropanol at 817 cm^−1^ due to symmetric C–O stretching of the molecules, and the same was also observed among the commercial HS. Moreover, ethanol-based HS showed a peak at 1050 cm^−1^ due to asymmetric C–O stretching. In contrast, isopropanol-based HS presented symmetric C–C stretching vibration and asymmetric C–O stretch at 948 cm^−1^ and 1126 cm^−1^, respectively. The peak at 1650 cm^−1^ in HS spectra indicated the characteristic –OH bending mode of water.

**Table 1:** Assignment of chemical bonds in FTIR spectrum (Griffiths 2006; Smith 2017a, 2017b; Anjos et al. 2020; Khalique Ahmed et al. 2010)

Wavenumber (cm^−1^)	Assignment	Class and group
680–620	C–O–H bending	Alcohol
817	C–O stretching	Isopropanol
881	C–O stretching	Ethanol
950	CH_3_–C–CH_3_ stretching	Secondary alcohols
1050	C–O stretching	Primary alcohol
1086	C–O stretching	Primary alcohol
1126	C–O stretching	Secondary or tertiary alcohol
1309	O–H bending	Secondary alcohol
1650	HOH bending	Water
2900	CH stretching	Glycerol 2883-Symmetric stretching 2935-Asymmetric stretching
3300	OH stretch	Alcohols and phenols
3400	Hydrogen bonded OO–H stretch	Hydrogen peroxides

**Fig. 2:**
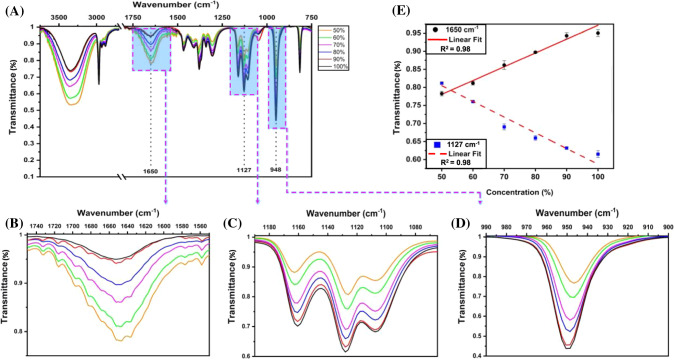
FTIR spectra of in-house prepared HS. **A** the spectra of in-house prepared HS with varying concentrations of isopropanol. **B**–**D** The variation of the FTIR transmittance intensity at 948 cm^−1^ and 1127 cm^−1^ as a function of isopropanol concentration, whereas at 1650 cm^−1^ as a function of water, has been shown. **E** Integrated linear plots with varying alcohol and water concentration. The data are plotted as a function of transmittance intensity. The statistical values are estimated for each recombination path

Along with the confirmation, quantifying the amount of alcohol is undoubtedly essential to meet HS’s quality control and assurance process. FTIR measurements were undertaken for in-house prepared HS with various concentrations of alcohol (ethanol and isopropanol) from 50%-100%. Figure 1C shows the combined FTIR spectra of the HS with varied ethanol concentrations. Three prominent peaks were observed at 881 cm^−1^, 1046 cm^−1^, and 1650 cm^−1^, which correspond to symmetric and asymmetric C–O stretch of ethanol, and O–H bending mode of water. It was also observed that the transmittance intensity changes with an increase in ethanol quantity. The transmitted intensity for the peaks 1046 cm^−1^ and 1650 cm^−1^ was observed to gradually decrease and increase, respectively, with increased ethanol concentration (Fig. 1D and E). Moreover, the transmittance of the peak at 881 cm^−1^ was also found to decrease progressively with an increase in ethanol concentration (Fig. 1F). The linear plots considering the variation of intensities in 1046 cm^−1^ and 1650 cm^−1^, as shown in Fig. 1G, suggest a good correlation (*R*^2^ = 0.98 and 0.98, respectively) to determine the difference between ethanol and water concentration using FTIR. In-house prepared HS with varied isopropanol amounts were also examined, and the combined spectra are shown in Fig. 2. Three prominent peaks were observed at 948 cm^−1^, 1127 cm^−1^, and 1650 cm^−1^,  corresponding to symmetric C–C stretching vibration, asymmetric C–O stretching of isopropanol, and O–H bending mode of water, respectively. The transmitted intensity at 948 cm^−1^ and 1127 cm^−1^ was observed to decrease with increased isopropanol concentration; conversely, the intensity at 1650 cm^−1^ increased as the water amount in the sample reduced. The variability in isopropanol and water concentrations, as presented by the varying intensity at 1127 cm^−1^ and 1650 cm^−1^ could be easily determined with a high correlation (*R*^2^ = 0.98 and 0.98) as shown in Fig. 2E.

The alcohol vibration modes of the HS at specific bands showed a left shift in the peaks with the variation in the amount of alcohol. The vibrational bands were blue-shifted upon varying the quantity of water to formulate HS with different alcohol concentrations. The higher vibrational frequency indicates a stronger covalent bonding. But as the alcohol amount decreases, it causes the weakening of the intermolecular interactions between the molecules, thereby showing the blue-shift (Corsetti et al. 2015). The shift was observed for the symmetric and asymmetric C–O stretching (881 cm^−1^ and 1046 cm^−1^) of ethanol as well as for symmetric C–C stretching vibration and asymmetric C–O stretching of isopropanol (948 cm^−1^ and 1127 cm^−1^). Based on the assessment of commercial HS as well as in-house prepared HS samples, it could be devised that assessed alcohol amount in most of the commercial HS samples falls in the range as mentioned in the labeling. However, in the case of samples B and E, the labeling mentioned the presence of 95% alcohol. But the intensity of characteristic peak at 881 cm^−1^ for B was found to be lower than E, highlighting that the quantified amount of alcohol was less. This proves that the study could be effectively used for quantifying the amount of alcohol in HS.

**Fig. 3:**
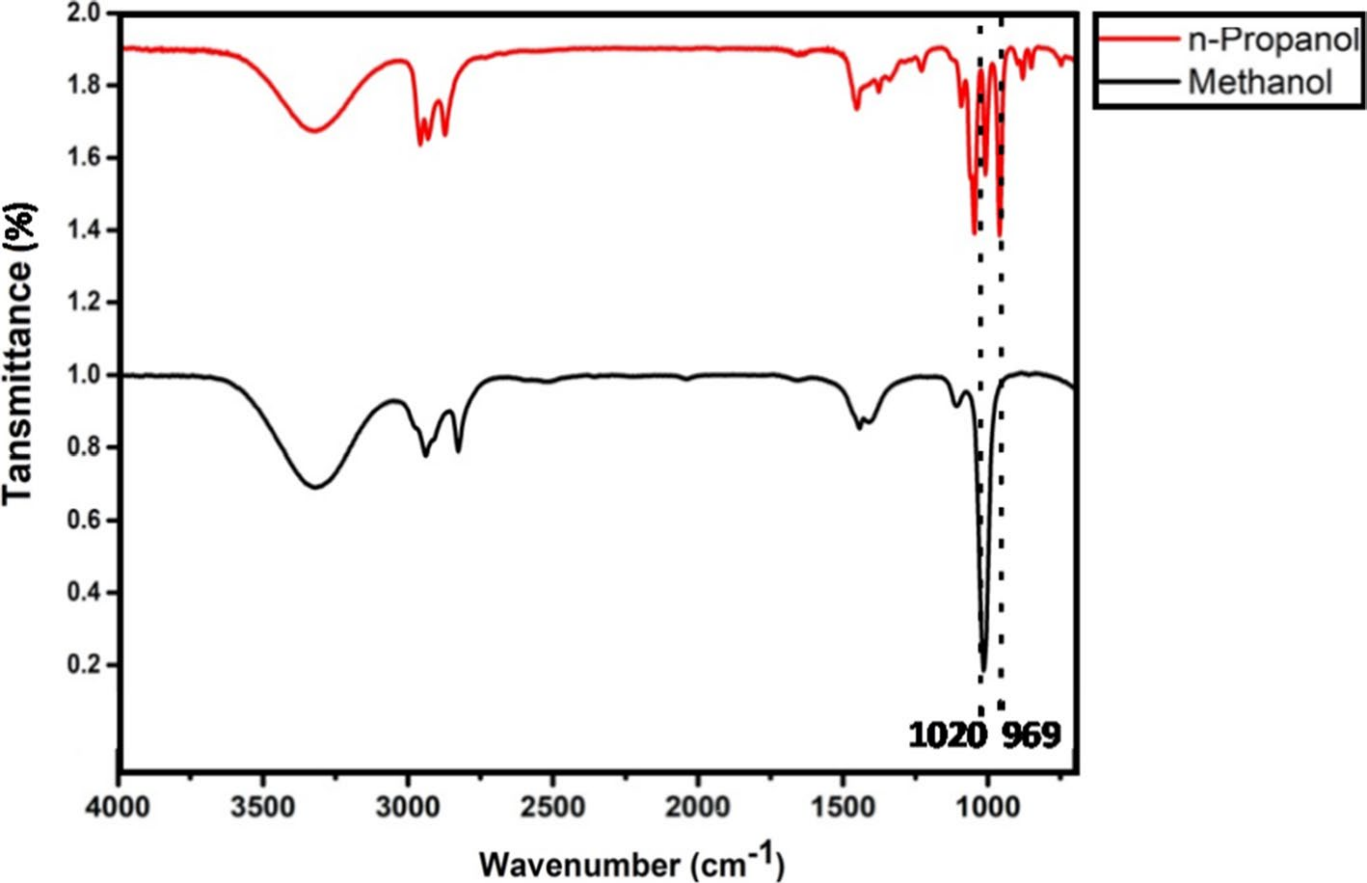
FTIR spectra of n-Propanol and methanol with their characteristic peaks highlighted

On the other hand, alcohol-based HS are often adulterated with methanol, as it is a cheaper alternative and known to have severe adverse effects on the human body (Chan and Chan 2018). Several companies also use n-propanol other than the recommended isopropanol to formulate HS. Even though both n-propanol and isopropanol do not show any severe adversaries on human bodies (Tasar et al. 2021), it is important to rule out the possibilities of alternative alcohol sources in HS. In this regard, FTIR spectra of methanol and n-propanol were also acquired independently and shown in Fig. 3. Their characteristic peaks were observed at 1020 cm^−1^ due to C–O stretching and 969 cm^−1^ due to C–C–C–O stretching, respectively, which helped in easy discrimination of these two alcohols from ethanol and isopropanol. The absence of a peak at 950 cm^−1^ in primary alcohol, particularly methanol, also helped distinguish the type of alcohol present in HS. Comparing these FTIR peaks with the commercial HS in Fig. 1 confirmed that the samples assessed in the study are devoid of methanol and n-propanol as formulates. Likewise, the presence of alcohol degrading agents such as methyl ethyl ketone and methyl isobutyl ketone in HS undermines its role of disinfection. FTIR spectroscopy is shown to identify these compounds based on peaks at 1710 cm^−1^ due to the C=O stretching of the ketone (Infrared Spectra of Some Common Functional Groups 2020; Samantha 2005). Hence, confirming the ability of FTIR spectroscopy in determining contaminants in HS and also their absence in the studied samples. The FTIR spectra of different raw materials, including glycerol (C_3_H_8_O_3_), water (H_2_O), and hydrogen peroxide (H_2_O_2_), were also recorded and are shown in Figure S1 (see supplementary information).

Even though FTIR spectroscopy provides a simple, fast, and accurate HS analysis process, different overlapping functional groups are often observed. Thus, an alternative spectroscopic method based on Raman scattering, which can provide molecular fingerprints for various chemical compoundsis further employed.

### Raman Spectroscopy

**Fig. 4:**
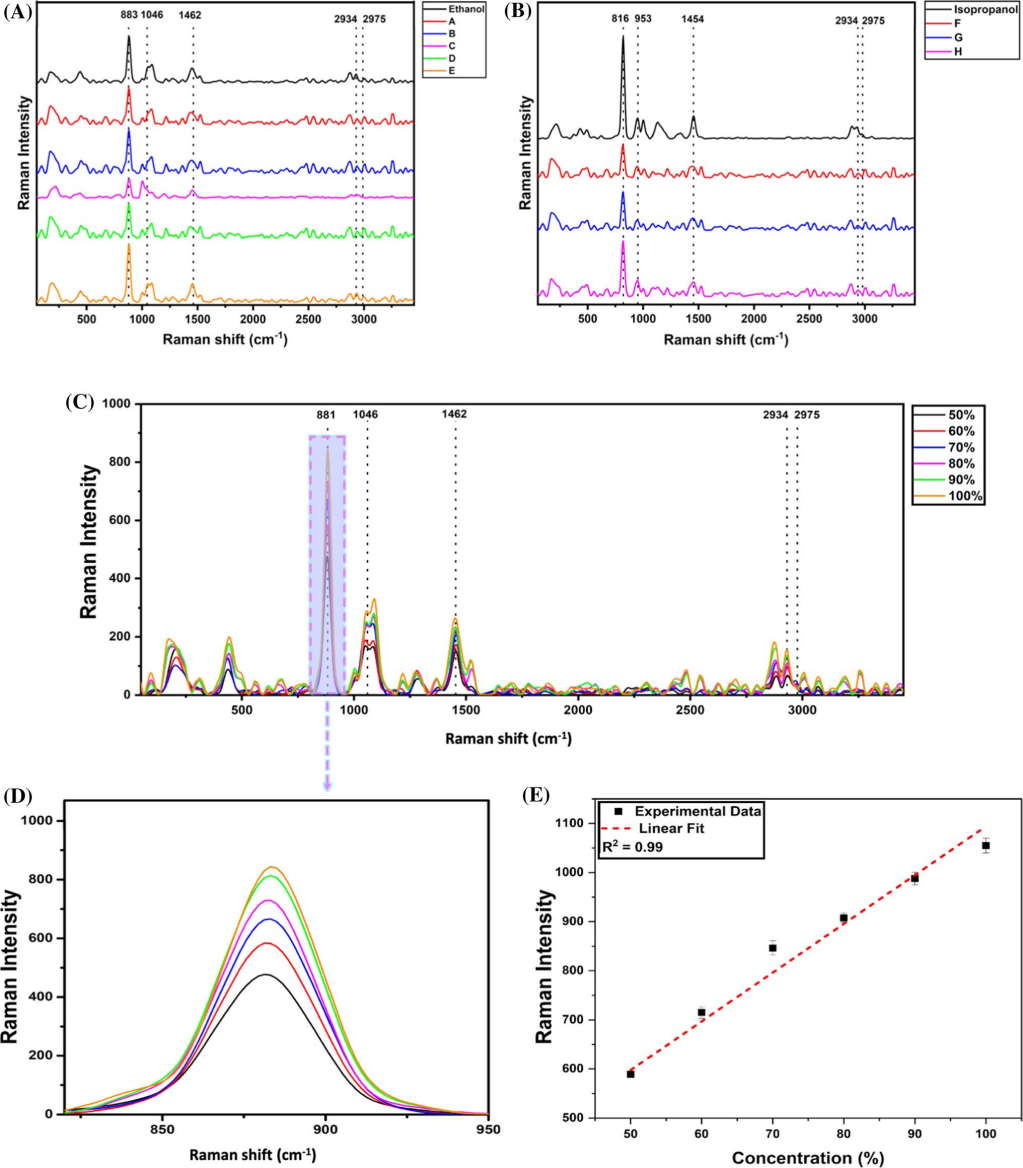
Raman spectra of various brands of HS; **A** ethanol-based HS and **B** isopropanol-based HS. The major spectral features associated with alcohols and water are indicated. **C**–**D** Raman analysis of in-house prepared HS. **C** shows the spectra of in-house prepared HS with various concentrations of ethanol. **D** The variation of the FTIR transmittance intensity at 883 cm^−1^ as a function of ethanol. **E** Integrated linear plots with varying alcohol and the data are plotted as a function of transmittance intensity. The statistical values are estimated for the linear fit

Raman spectroscopy is a powerful, non-destructive spectroscopic method that uses monochromatic light in visible or near-infrared regions to determine the compound’s vibrational modes (Krishnan and Shankar 1981). Since the Raman vibrational modes of a compound are more specific, this type of spectroscopy generates unique fingerprint spectra. In the study, Raman spectra of various HS brands were acquired and analyzed. Figures 4A and B show the Raman spectra of ethanol and isopropanol-based commercial HS respectively. These could be distinguished into two groups due to specific Raman fingerprints at 883 cm^−1^ (ethanol) and 816 cm^−1^ (isopropanol). Table 2 shows the Raman vibrations observed for various ingredients of HS. Further, HS samples were also assessed for various other constituents as mentioned in table S1. The majority of the commercial samples contained vitamins and plant extract in form of essential oil. The presence of a peak at 1582 cm^−1^ represented the presence of vitamin E in the samples. As essential oils contain alkenes of different lengths, the C=C stretch around 1670 cm^−1^ is an important assessment for detecting the presence of oil in the sample and this peak was observed in most of the commercial HS samples except in Raman spectra of alcohols. Likewise, the presence of acrylate could be assessed based on the detection of acrylate double bond at 1407 cm^−1^. Besides these commonly used raw ingredients, the presence of various additional chemical compounds used in commercial HS, as mentioned in Table S1, can also be determined using Raman spectrometry. The Raman-specific vibrational modes of these chemicals are highlighted in Table S3, and these modes can be used for qualitative assessment of HS.

**Table 2:** Assignment of Raman fingerprints (Ramírez-Cedeño et al. 2012; Emin et al. 2020; Boyaci et al. 2012; Gelder et al. 2007; Hickstein et al. 2018)

Wavenumber (cm^−1^)	Assignment	Class and group
816	C–C–O symmetric stretching vibration modes	Isopropanol
878	O–O stretching	Hydrogen peroxide
883	C–C–O symmetric stretching vibration modes	Ethanol
1046	C–O scaling modes	Ethanol
1104	CCO skeleton stretching	Ethanol
1454	CH_3_ anti-symmetric vibration	Isopropanol
1462	CH_3_ anti-symmetric vibration	Ethanol
1465	C–H deformation	Glycerol
2934	CH_2_ asymmetric stretching vibration modes	Alcohol
2975	CH_3_ asymmetric stretching vibration modes	Alcohol

**Fig. 5:**
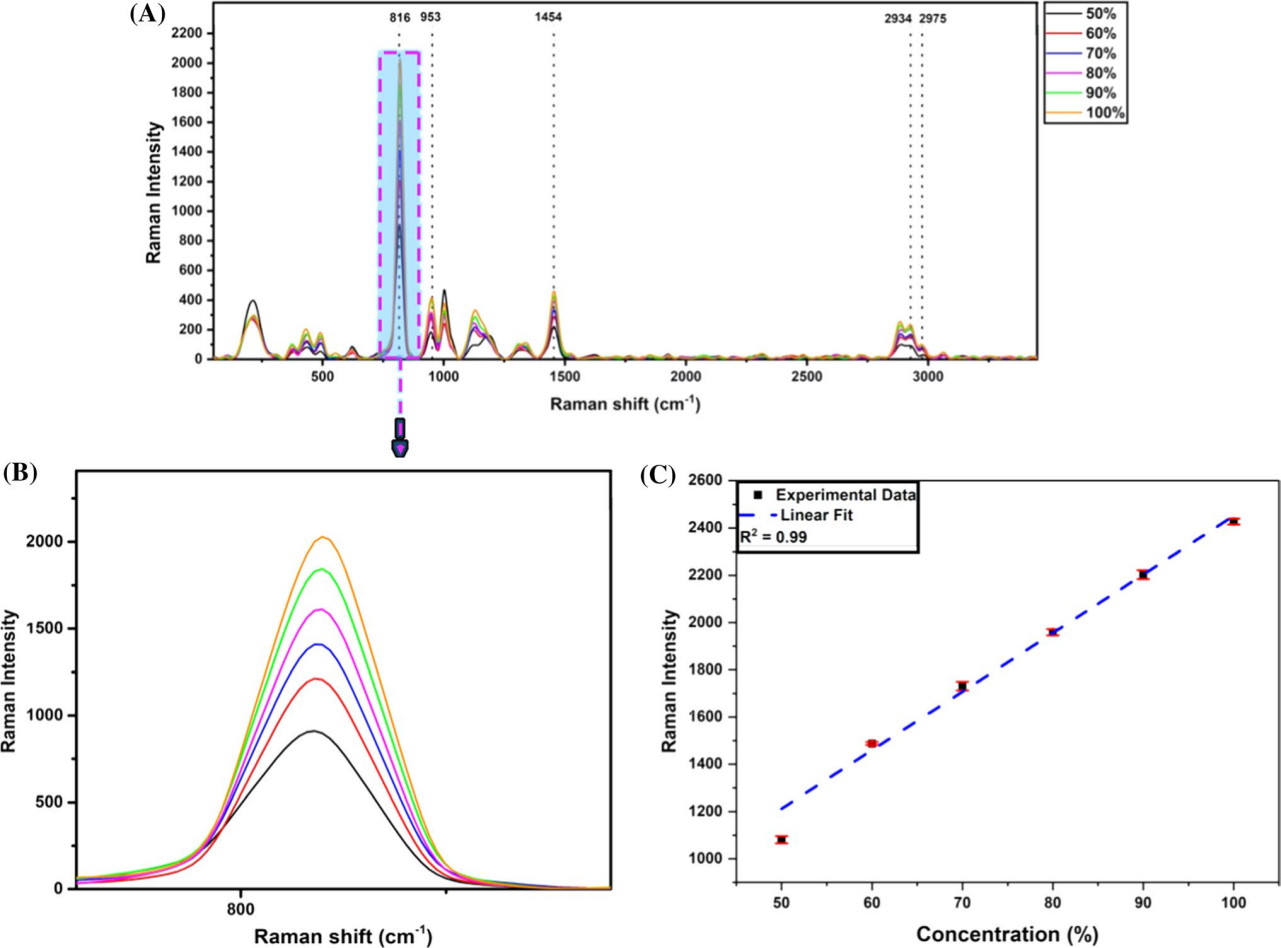
Raman spectra of in-house prepared HS. **A** shows the spectra of in-house prepared HS with various concentrations of isopropanol. **B** The variation of the FTIR transmittance intensity at 818 cm^−1^ as a function of isopropanol. **C** Integrated linear plots with varying alcohol and the data are plotted as a function of transmittance intensity. The statistical values are estimated for the linear fit

Further, ethanol and isopropanol-based HS were prepared in-house according to the WHO protocol with varying ethanol and isopropanol concentrations and are subjected to Raman spectroscopy. Raman spectra of ethanol-based HS with alcohol amount ranging from 50 to 100% are shown in Fig. 4C. The fingerprint Raman intensity at 883 cm^−1^ as in Fig. 4D corresponding to C–C–O symmetric stretching vibration modes in ethanol increases with an increase in alcohol concentration. The linear plot can also confirm that Raman spectroscopy identifies the ethanol-based HS with varying alcohol amounts as represented in Fig. 4E with a high linear correlation (*R*^2^ = 0.99). It had also been observed that there was a shift in the peak at 883 cm^−1^ with the increase in ethanol concentration. Likewise, the amount of isopropanol in HS can be quantified and assessed for various samples as shown in Fig. 5A. HS with varying alcohol concentrations were analyzed, and the peak at 816 cm^−1^ was observed to be the fingerprint peak related to isopropanol due to the C–C–O stretching in the molecule and was found to vary in intensity with a change in concentration, as represented in Fig. 5B. And the same is confirmed with the plot (*R*^2^ = 0.99) shown in Fig. 5C.

Similarly, the shift was also observed for the ethanol and isopropanol specific peaks, indicating the strengthening of the intramolecular hydrogen bonding with increased alcohol content. Apart from these prominent peaks, various other peaks can also be inferred from the spectra in Figs. 4 and 5, which have been highlighted along with their assignment in Table S3. It was observed that ethanol shows C–O stretching at 1046 cm^−1^ and CH_3_ rocking at 1079 cm^−1^, indicated by two small bands in the same region as seen in the spectra. Anti-symmetric vibration of CH_3_ in ethanol is also observed at 1462 cm^−1^. Furthermore, CH_2_ asymmetric stretching vibration modes at 2934 cm^−1^ and CH_3_ asymmetric stretching vibration modes at 2975 cm^−1^ were also observed. The Raman spectra of different raw materials were acquired as shown in Figure S2 (see supplementary information). Similar peak positions are also observed in Fig. 3. It was observed that glycerol shows a characteristic peak at 1465 cm^−1^ due to the C–H deformation in the molecule. The characteristic peak of hydrogen peroxide was observed due to O–O stretching at 878 cm^−1^, which is seen clearly in the raw H_2_O_2_ spectra in Figure S2 (see supplementary information) but found to be merged in HS’s spectra (in Fig. 3, 4, and S3) as it is present in shallow amounts.

**Fig. 6:**
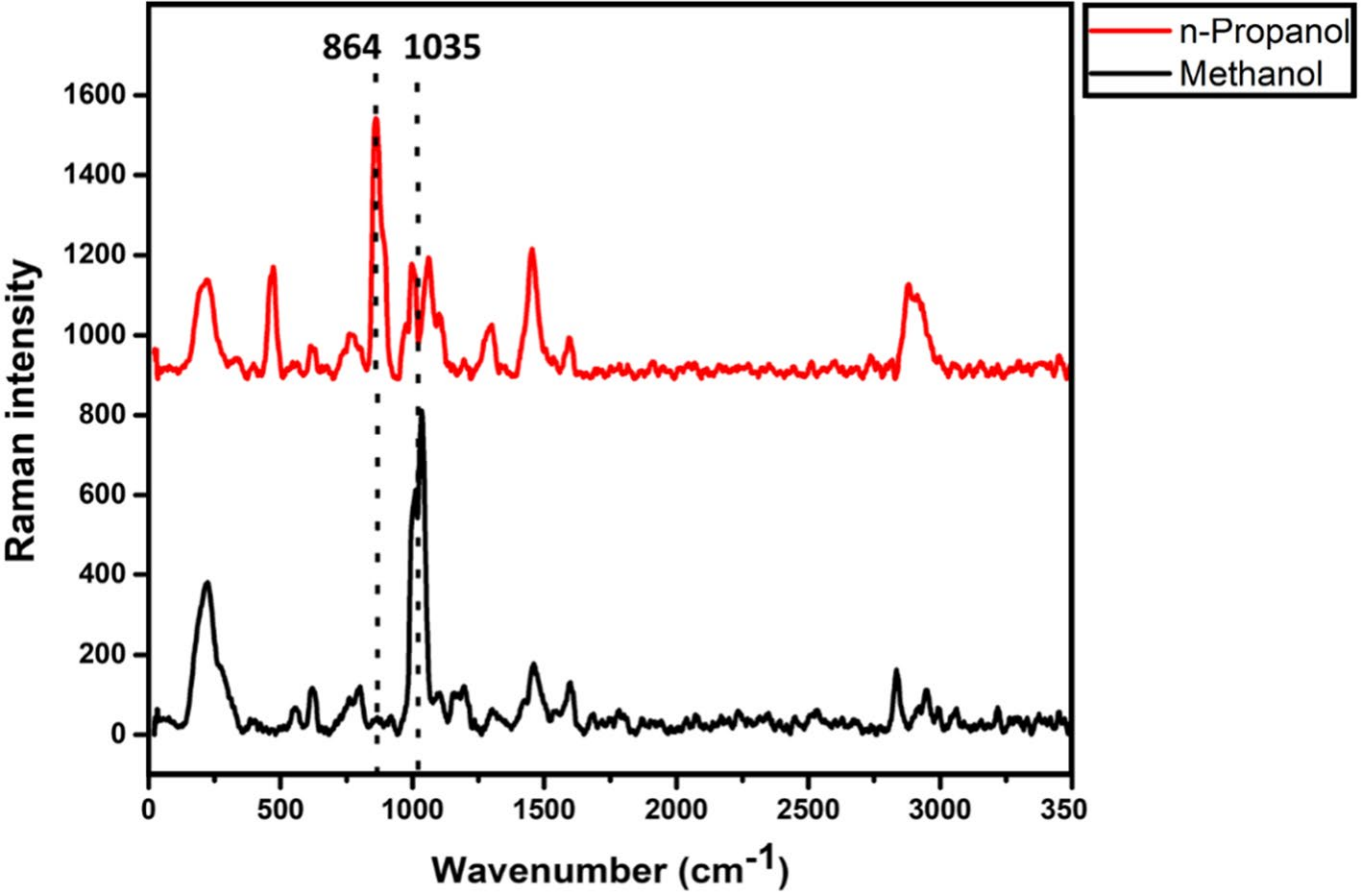
Raman spectra of n-Propanol and methanol with their characteristic peaks

As Raman spectroscopy has shown its ability to generate compound-specific spectra, it can discriminate among the alternative alcohols used in HS as adulterants. Raman spectra of methanol and n-propanol were acquired as depicted in Fig. 6 and analyzed to find the characteristics of Raman peaks at 1035 cm^−1^ due to C–O stretching (Fiume et al. 2013) and 864 cm^−1^ due to structural C–C–O stretching, respectively (Wu et al. 2017). These peaks do not fall in the same range as ethanol (883 cm^−1^) and isopropanol (816 cm^−1^) and facilitate easy identification of the type of alcohol in HS. Further, contaminants such as methyl ethyl ketone and methyl isobutyl ketone, which are known to denature alcohols, can be present in HS, making them toxic to use, foul-smelling, and ineffective in sanitization. Raman spectroscopy can detect these compounds’ presence based on the characteristic peak at 761 cm^−1^ from C_2_-C_3_ and C_3_-C_4_ stretch vibration for methyl ethyl ketone (Suci et al. 2001). Whereas, methyl isobutyl ketone can be identified based on its Raman-specific vibrational modes at 2920 cm^−1^ (symmetric CH_3_ vibration) and 2960 cm^−1^ (asymmetric CH_3_ vibration), providing the argument that this type of spectroscopy can be used for assessing the alcohol contaminants in HS.

Vibrational spectroscopic methods like FTIR and Raman spectroscopy efficiently demonstrated their ability to quantify alcohol and water content in the HS as well as the presence of different chemical ingredients in them. However, a downfall was observed in detecting and distinguishing various additives such as medicinal tree extracts and perfumes based on FTIR and vibrational modes of such compounds (Emin et al. 2020). Therefore, fluorescence and UV–Vis absorbance spectroscopy can be used to overcome this limitation. The commercial HS samples and the in-house were also assessed using UV–Vis absorbance and fluorescence spectroscopy.

### UV–Visible absorption Spectroscopy

**Fig. 7:**
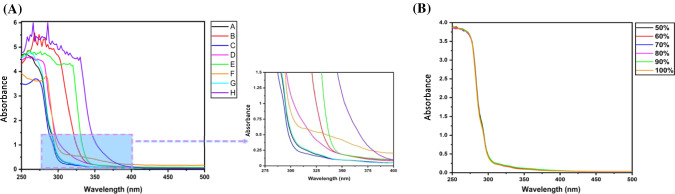
UV–Vis absorbance spectra of **A** different brands of HS for the 250–500 nm spectral region. The zoomed-in region shows the absorbance in the spectral region of 275–400 nm. **B** in-house prepared ethanol-based HS.

UV–Vis absorption spectroscopy was performed to determine the peak absorption wavelength of different constituents of various HS in an attempt to assess them, and the resulting spectra are shown in Fig. 7. All HS are made of various chemical components, and they tend to absorb photons in different spectral ranges. The absorbance of commercial HS was observed between 250–500 nm, as shown in Fig. 7A. It was found that brands A, C, and G show absorption wavelengths less than 300 nm whereas the remaining brands (B, D, E, F, and H) are within 325 to 400 nm. Sample A contained chlorhexidine gluconate, which had a peak absorbance at 259 nm, while C contained propylene glycol, which had a peak absorbance at 260 nm. The presence of tocopherols and phenolic substances is determined by absorbance at 325 nm. Plant-based compounds were found in B, D, E, F, and H, and most of these compounds were in the form of oils containing carotenoids or chlorophyll, with absorbance in the 400–500 nm range. Furthermore, the absorbance of in-house prepared HS with varying ethanol concentrations was recorded. No dissimilarity was observed within the absorption spectrum with the change in alcohol and water amount, as shown in Fig. 7B. Hence, proving that variation in concertation of raw ingredients does not change HS’s absorbance. Additionally, the absorbance of raw ingredients was also examined separately and represented in Figure S3 (see supplementary information). Hence, such spectroscopy can be used as an alternative method for the quality control assessment of HS. Besides, it is generally discouraged to add such fragrances due to the risk of allergic reactions.

### Fluorescence Spectroscopy

**Fig. 8:**
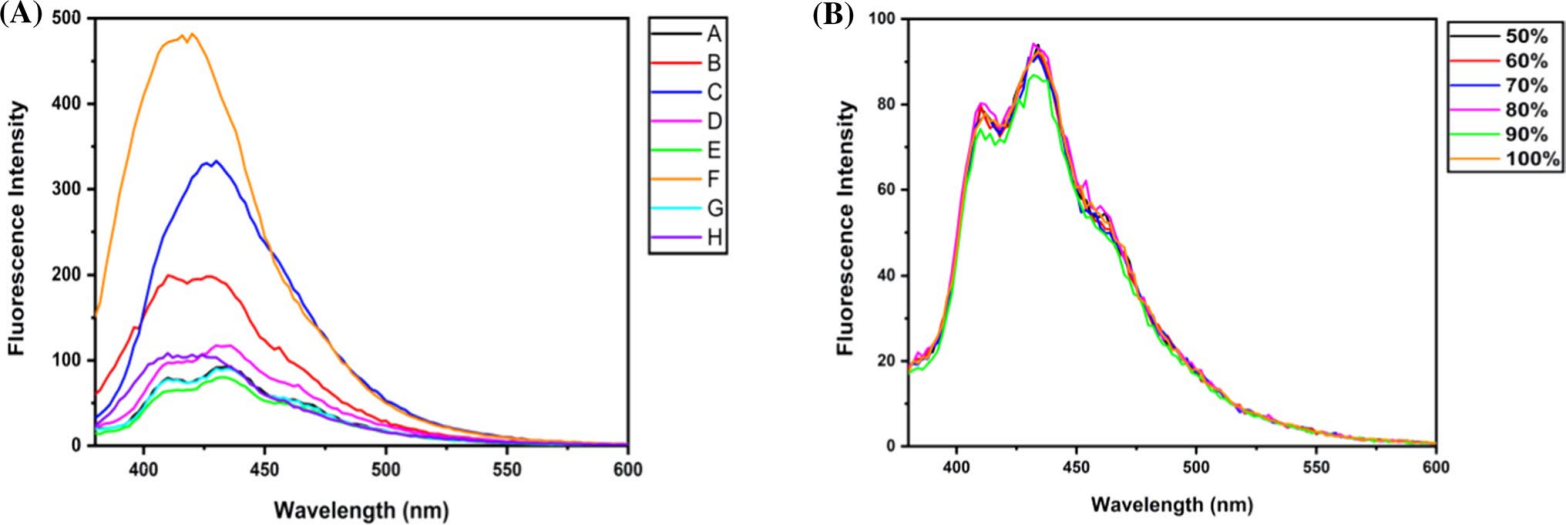
Fluorescence spectra of **A** various brands of HS, and **B** in-house prepared HS with various concentrations of ethanol are shown with an excitation wavelength at 350 nm

FTIR and Raman spectroscopy provided information about the functional groups present in HS whereas fluorescence spectroscopy was used to identify various types of additives in HS, which may be fluorescent. Various excitation wavelengths were used to measure the fluorescence intensities of HS. However, the highest fluorescence was observed for 350 nm excitation wavelength. Fluorescence spectra of commercial HS with an excitation wavelength of 350 nm are represented in Fig. 8. It was observed that brand F showed the highest fluorescence with a strong central peak at ~ 425 nm, followed by C and B. This peak mainly raised from the glycine present in H whereas in C it was due to the acrylate present in their formulation. Multiple emission peaks were observed in A, D, E, G. Previous studies have shown that most chemical compounds like triethanolamine and cyclic aromatic, which are fluorogenic, are added to cosmetics and HS. These ingredients are harmful to the skin and can cause local irritation. In addition, natural raw ingredients such as Vitamin E, Vitamin B3, and extracts of various medicinal plants that are added to HS to increase their esthetic effect and have intrinsic fluorescence properties. Therefore, these compounds can also be confirmed with ease using fluorescence spectroscopy, as indicated in Table S3. As the medicinal plant oil extracts contain carotenoids, fluorescence peaks in the range of 400–500 nm were observed. Similarly, the presence of Vitamin E in the HS samples can be assessed based on the presence of a peak in the range of 390–440 nm. Despite the presence of medicinal plant extract in the HS samples, no chlorophyll-related signal (at about 670 nm) was seen, indicating that these extracts may be present in a refined form devoid of chlorophyll or may not be present at all. Additional studies with chemical standards can be conducted to validate such instances.

Fluorescence spectroscopic analysis was also performed for the various in-house prepared HS with varying concentrations of alcohol. It was observed that with the increase in ethanol amount in HS, the fluorescence spectra were unchanged, as shown in Fig. 8B. Hence, the change in alcohol concentration or type of alcohol in HS does not affect the fluorescence signal. Thus, it can be enumerated that the fluorescence signals rise from the additives such as perfumes or tree extracts. Also, the fluorescence spectra of different raw ingredients used to prepare in-house HS are shown in Figure S4 (see supplementary information). It could be observed from Fig. 6 that the fluorescent emissions at 410 nm and 430 nm showed the most variation among the different HS samples. It was further explored to observe that these peaks in commercial samples showed more variation compared to in-house prepared ones as shown in Figure S5 (see supplementary information). Thus, fluorescence and UV–Vis absorbance spectroscopy were not able to distinguish the alcohol amount variation in HS. However, it only detected the presence of additives like plant extracts and vitamins.

## Conclusion

In this work, different spectroscopic techniques were applied to characterize commercial HS and were compared with in-house prepared HS to determine each technique’s efficiency. FTIR and Raman spectroscopy elucidated their role in quantitative and qualitative identification of the HS's chemical compositions. FTIR spectroscopy confirmed the presence of ethanol or isopropanol in HS based on the presence of characteristic peaks. Further, the amount of water content in the sanitizer could also be quantified based on the –OH bending peak’s intensity. Additionally, other HS ingredients, such as oxidizing agent (H_2_O_2_) or humectant (glycerol), could be identified with FTIR spectroscopy. Similarly, Raman spectroscopy detected different constituents’ present in HS based on the characteristic vibration of the bonds, specific to the chemical types. Therefore, these two spectroscopic techniques provided alternatives in probing and characterizing the different constituents of the HS and determining the type of alcohol and alcohol denaturing agent.

Moreover, commercial spectroscopic instruments’ sensitivity can be a drawback for such studies as the resolution and detection limit of the device are vital for the accurate recognition of minute contaminants in HS. Decomposing spectroscopic data and testing the various HS ingredient’s analytical standards would have provided better assessments, which was absent in the study. However, spectral analysis methods such as excitation-emission matrix can be employed in the future so that more information can be mined from the recorded spectra. Various statistical tools for easy interpretation of large spectral datasets can also be incorporated along with machine learning techniques for automated and rapid HS assessment. This will help in the development of a portable multi-modal analytical system for HS’s comprehensive assessment, which can be simple, rapid, and cost-effective.

## Supplementary Information

Below is the link to the electronic supplementary material.

Supplementary file1 (DOCX 2671 kb)

## Data Availability

The data can be accessed on request from the authors.\
