# Supplementary material for: Spectroscopic methods for assessment of hand sanitizers
Source: Chem Zvesti. 2022 Apr 25;76(8):4907–18. doi: 10.1007/s11696-022-02208-x (PMC9035981; doi:10.1007/s11696-022-02208-x)
Supplement: Supplementary file 1 — Supplementary file1 (DOCX 2671 kb) [file 11696_2022_2208_MOESM1_ESM.docx]

**SUPPLEMENTARY INFORMATION**

**Spectroscopic methods for assessment of hand sanitizers**

Soumyabrata Banik^1^, Sindhoora Kaniyala Melanthota ^1^, Anjana Anandan Vannathan^2^, Krishna Kishore Mahato^1^, Sib Sankar Mal^2,*^, Nirmal Mazumder^1,*^

^1^Department of Biophysics, Manipal School of Life Sciences, Manipal Academy of Higher Education, Manipal, Karnataka, India-576104

^2^Materials and Catalysis Lab, Department of Chemistry, National Institute of Technology Karnataka, Surathkal, Karnataka, India-575025

***Corresponding authors:** nirmaluva@gmail.com & malss@nitk.edu.in

Hand sanitizers (HS) were the most commonly used disinfectant during the recent pandemic and have been found to significantly lessen the impact of disease propagation. With the increasing demand, qualitative and quantitative assessment of HS is important [1]. We have demonstrated the examination of HS with Raman and FTIR spectroscopy techniques and showed that both methods are efficient in accurately determining the different ingredients as well as quantifying the amount of alcohol. However, these HS also contains different constituents like perfumes and tree extracts which are often difficult to with the mentioned spectroscopic methods. Therefore, the additives in the HS samples were assessed using UV-Vis (Ultraviolet-Visible) absorbance and fluorescence spectroscopy.

**Table-S1: Composition of different alcohol-based hand sanitizers used in the study**

| **Brand Code** | **The composition as mentioned in the packaging of the HS** | **Alcohol type and its content** |
| --- | --- | --- |
| A | Ethyl-alcohol (70%), Chlorhexidine gluconate (0.5%) | Ethanol (70%) |
| B | Ethyl-alcohol (95%), Isoparaffin (62%), Isopropyl-alcohol (3%), Niacinamide (1%), Perfumed gel base | Ethanol (95%) |
| C | Ethyl-alcohol (72.34%), water, PEG/PPG Copolymer, Propylene Glycol, Acrylates, Tetrahydroxyproplyethylenediamine, perfume | Ethanol (72.34%) |
| D | Ethyl-alcohol (60%), and water along with extracts of Coriander, Vetiver, Nutgrass, Spiked ginger lily, and Neem. | Ethanol (60%) |
| E | Ethyl-alcohol (95%), propylene glycol, Diethyl phthalate, tea tree leaf oil, lemon peel oil, *Azadirachta indica* leaf extract, *Ocimum santum* extract, glycine, acrylates, triethanolamine, perfume | Ethanol (95%) |
| F | Isopropyl alcohol (70%), Glycerin, triethanolamine, Carbopol 940, perfume, Neem extract, Aloe vera extract, Phenoxyethanol, water | Isopropanol (70%) |
| G | Isopropyl alcohol (75%), Glycerol (98%), H_2_O_2_ (30%), distilled water | Isopropanol (75%) |
| H | Isopropyl alcohol (80%), lime glycerin, anti-bacterial solvents, herbal oils, aloe vera, tulsi, vitamin E contents & Fragrance | Isopropanol (80%) |

**Table-S2: Composition of different alcohol-based hand sanitizers prepared in-house using WHO standard in the study**

| **Sample code** | **Raw materials (μL) (Total volume-10000 μL)** | | | |
| --- | --- | --- | --- | --- |
|  | **Alcohol (Ethanol/Isopropanol)** | **Water** | **Glycerol** | **H_2_O_2_** |
| 50% | 5000 | 4815 | 145 | 40 |
| 60% | 6000 | 3815 |  |  |
| 70% | 7000 | 2815 |  |  |
| 80% | 8000 | 1815 |  |  |
| 90% | 9000 | 815 |  |  |
| 100% | 9815 | 0 |  |  |

**FTIR Spectroscopy:**

The FTIR spectra of different raw materials, including glycerol (C_3_H_8_O_3_), water (H_2_O), and hydrogen peroxide (H_2_O_2_), were recorded and shown in Figure S2. Glycerol shows characteristic C-H stretching at 2900 cm^-1^ (2883 cm^-1^ for symmetric and 2935 cm^-1^ for asymmetric stretching), and the same peaks were also observed in the commercially available HS. The region 3420-3250 cm^-1^ shows the presence of O-H stretching in alcohols and phenols, whereas the 3400-3300 cm^-1^ shows hydrogen-bonded OO-H stretching in H_2_O_2_. Further, the FTIR characteristics bands of the different ingredients used in commercial HS (as mentioned in Table S1) have been highlighted in Table S3 based on available literature. This shows that FTIR spectroscopy can be used to identify different chemicals in HS and be implemented for the qualitative detection of such molecules.

**Figure S1:** FTIR spectra of the raw ingredients used to prepare in-house HS according to WHO protocol. The spectra related to H_2_O, glycerol, and H_2_O_2_ have been shown, highlighting their characteristic peaks.

**Raman Spectroscopy**


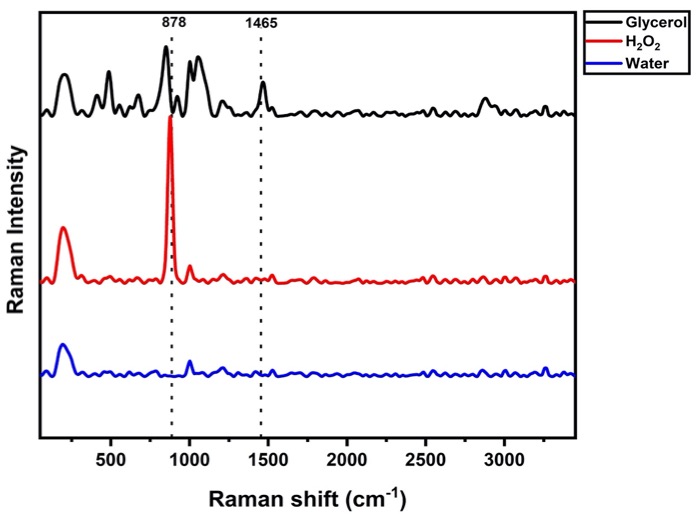


**Figure S2**: Raman spectra of the raw ingredients used to prepare in-house HS according to WHO protocol. The spectra related to H_2_O, glycerol, and H_2_O_2_ have been shown, and their characteristics Raman peaks have been highlighted.

A downfall is detecting various additives such as medicinal tree extracts and perfumes in samples based on vibrational modes of such compounds. Therefore, fluorescence and UV-Vis absorbance spectroscopy can be used to overcome this limitation. As many of the compounds may be present in a very minute amount, therefore often the sensitivity of the instrument used, and the limit of detection plays a salient factor for their identification. Thus, other spectroscopic methods that can detect the compounds based on their absorbance or fluorescence properties may prove useful [2, 3].

**UV-Visible absorption Spectroscopy**

Absorbance spectra were recorded for individual raw materials, shown in Figure S8, and compared to HS of different brands. Additionally, the absorbance wavelengths in the UV-Vis range of various formulates in commercial HS (as mentioned in Table S1) have been highlighted in Table S3. It can be enumerated from Table S3 that most of these formulants absorbed light in the UV region with a difference in their absorbance wavelength. This can be used for the qualitative assessment of HS and rapidly to determine the presence of such compounds.

**Figure S3:** UV-Vis absorbance spectra of the raw ingredients used for the preparation of in-house HS according to WHO protocol. The absorbance spectra related to ethanol, isopropanol, H_2_O, glycerol, and H_2_O_2_ have been shown for the 250 – 500 nm spectral region.

**Fluorescence Spectroscopy**


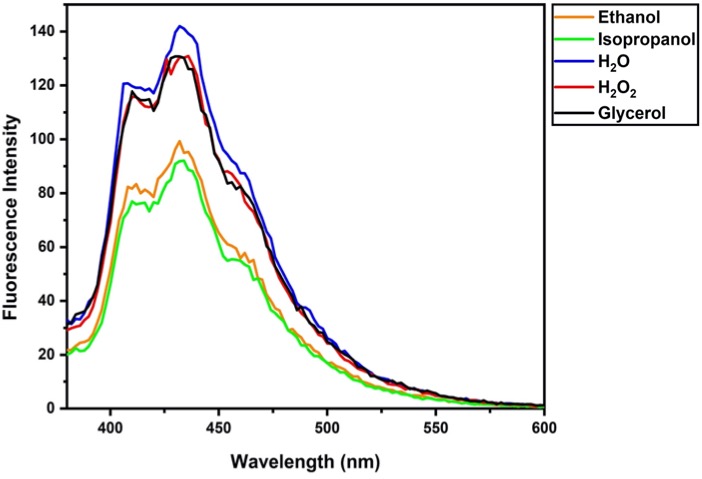


**Figure S4:** Fluorescence spectra of the raw ingredients used to prepare in-house HS according to WHO protocol. The fluorescence spectra related to ethanol, isopropanol, H_2_O, glycerol and H_2_O_2_ have been shown for the excitation at 350 nm. The emission spectral was recorded for the spectral region of 450 – 600 nm.

Two prominent fluorescence emission peaks at 410 nm and 430 nm were analyzed to understand the variation in fluorescence intensity among the commercial and in-house prepared HS samples. The variation in fluorescence intensity at these wavelengths has been shown in Figure S7. It can be inferred that with the change in the amount of ethanol among the in-house prepared HS samples, the variation in fluorescence emission intensities was found to be less than 5%. Whereas considering the same emission wavelength intensities, considerable variation among the commercial HS samples was observed, confirming the presence of additional ingredients and supplementing the results of Figure 6. Therefore, it can be suggested that the common fluorophores present in the samples get excited at 350 nm and the changes are due to the presence of fluorogenic ingredients in commercial HS. Thus, in this study, fluorescence spectroscopy has been used for fingerprinting purposes to detect the presence of any fluorogenic compounds in the HS. However, the device’s sensitivity plays a key role in such qualitative assessments and easy identification of compounds. With the rapid development of various modular spectroscopies in the market and numerous scientific publications demonstrating their uses, it can be emphasized that there is a vast possibility in the analytical assessment of everyday used products using fluorescence spectroscopy.

**Figure S5:** Graphs show the variation in fluorescence intensities with different concentrations of ethanol (A) and brands (B) of HS. The fluorescence intensities are plotted at emission wavelengths of 410 nm and 430 nm, respectively.

**Table S3: Spectral analysis of various additives in commercial HS [4-40]**

| **HS sample** | **Compound name** | **FTIR spectroscopy compound-specific peak wavenumber**  **(assignment)** | **Raman spectroscopy compound-specific peak wavenumber**  **(assignment)** | **UV-Vis absorbance spectroscopy compound-specific peak wavelength** | **Fluorescence spectroscopy compound emission peak wavelength** |
| --- | --- | --- | --- | --- | --- |
| A | Chlorhexidine gluconate | 1533 cm^-1^ and 1492 cm^-1^ (stretching of N-H bond) | 1585-1626 cm^-1^ (guanidine groups) | 259 nm | - |
| B | Isoparaffin | - | - | - | - |
|  | Niacinamide | 1690 cm^−1^ (CO stretching vibrations) and 3520 cm^−1^ (N–H stretching vibrations) | 1031 cm^-1^ | ~260 nm /~325 nm | 430 nm |
|  | Perfumed gel base | Distinct perfume base constituent not mentioned in the label of the product | | | |
| C | PEG/PPG Copolymer | The peaks around 2888, 2920, and 2888 cm^−1^ (are attributed to the alkyl chain of polymer); the bands at 1342 and 1100 cm^−1^ (are due to C-H bending and C-O stretching vibration, respectively); and the band at 1242 cm^−1^ (C-H twisting vibrations ) –PEG  Absence of 3560 and 3390 cm^-1^ peaks indicate PEG-PPG coexist in solution | 2938, 2886, and 2843 cm^−1^ (vibrations of alkyl chains );  1478 and 1442 cm^−1^  (bending mode of the C-H group) | 450 nm | - |
|  | Propylene Glycol | 3350 cm^-1^ | 838 cm-1 | 260 nm |  |
|  | Acrylates | 1410 cm^-1^ (nCOO- stretching) | 1407 cm^-1^ and 1635 cm^-1^ (The acrylate  double bond) | 212 nm | 465 nm |
|  | Tetrahydroxyproplyethylenediamine | 2970 cm^-1^ | 2930cm^-1^ | - | - |
| D | Extracts of Coriander, Vetiver,  Nutgrass,  Spiked ginger lily,  Neem. | - | - | - | - |
| E | Propylene glycol | - | | | |
|  | Diethyl phthalate | 1730 cm^-1^ | 2940 cm^-1^ | 275 nm | - |
|  | Tea tree leaf oil | - | - | - | - |
|  | Lemon peel oil | - | - | - | - |
|  | Azadirachta indica leaf extract | 1642 cm^-1^ corresponds to C=O group | - | ~ 310 nm | ~ 450 nm |
|  | Ocimum santum extract | - | - | - | - |
|  | Glycine | carbonyl stretching group between 1730 and 1750 cm^−1^ for amino acids | 900 cm^-1^ for carboxy group vibrational mode | 216 nm | 452 nm |
|  | Acrylates | - | | | |
|  | Triethanolamine, (Toxic) | 3350 cm^-1^ | 1154 cm^-1^ | ~210 nm | 508 nm |
| F | Glycerin | As mentioned in Table 1 & 2 | | | |
|  | Triethanolamine | - | | | |
|  | Carbopol 940 | 1712.79, around 1454, around 1414, around 1245, around 1115, and around 801 cm-1 | 523.9 cm^-1^ (bending vibration of C-C-O group)  876.8 cm^-1^ (stretching vibration of C-O-C for acrylates and carboxylic acid)  1366.5 cm^-1^ (symmetric vibration of O-C-O of acids) |  |  |
|  | Neem extract | - | | | |
|  | Aloevera extract | - | | | |
|  | Phenoxyethanol | 1250 cm^-1^ | 1000 (vs)  1010 cm^-1^ | 220 nm |  |
| H | Vitamin E contents | 900–1077 cm^-1^ (C=O stretching vibration); 1205 cm^-1^ (C=C formation); 2925 cm^-1^ (C-H alkanes group) | 1582 cm^-1^ and 1615 cm^-1^ | 219 nm | 390-440 nm |
|  | Aaloe vera, | 1634 and 1418 cm^-1^ (asymmetrical and symmetrical -COO stretching of carboxylate compounds) | - | ~ 210 nm and ~ 550 nm (peak) | - |
|  | Ocimum santum extract (tulsi) | 1635 cm^−1^ (groups C=C), 2100 cm^−1^(C≡C group) and 3300 cm^−1^(amine N-H/O-H vibration stretch) | ~ 2000 cm^-1^ | ~ 275 nm | - |

Even though FTIR and Raman spectroscopy are both vibrational types of spectroscopy, they are comparatively different in terms of their ability to detect different molecules. FTIR detects a molecule based on the absorbance of infrared radiation by it, and this absorbance depends on different states of the molecules such as stretching, bending, etc. FTIR spectroscopy is a more advantageous detection method if the ingredients in the solution are fluorescent, have strong dipole moments. While detecting ethanol and isopropanol, which have the difference of just one carbon in their chains, similar peaks are detected for both the molecules using FTIR. Some chemical bonds may not be detected by FTIR spectroscopy, and for it, another method such as Raman spectroscopy. As Raman spectroscopy is based on the scattering of light from different molecules, it can detect those bonds that are not measured by FTIR spectroscopy. Raman is useful in detecting symmetric molecules/bonds, investigating carbon bonds in aliphatic and aromatic rings, detecting bonds with weak dipoles are important and low-frequency modes. as shown in Table S4. There could be similar bonds that can be detected, with both FTIR and Raman spectroscopy. However, unique fingerprints can also be identified by either of the methods. These regions are very useful while detecting chemical ingredients that are similar to ethanol or isopropanol or even in case they react with the latter. The HS also contains perfumes and additives such as medicinal tree extracts that are difficult to identify using FTIR and Raman. Therefore, fluorescence and UV-Vis absorbance spectroscopy can be used to overcome this limitation. The study had the advantages of incorporating different spectroscopic methods simultaneously, which provided the edge in accurately determining the quality and quantity of HS ingredients. Incorporating Raman with FTIR spectroscopy is further useful as Raman is a more robust analytical tool in determining different constituents in the samples, which might be missed in FTIR spectra. The use of simple statistical analysis makes it easier for many users to use them without advanced knowledge. The methods used in this study are rapid, easy-to-use, and can be performed with higher accuracy at a much lesser cost.

**Table S4: Comparison of the bonds detected in ethanol and isopropanol by FTIR and Raman spectroscopy**

|  | **Ethanol** | **Isopropanol** |
| --- | --- | --- |
| **FTIR-specific bonds (cm^-1^)** | 1086 cm^-1^ - C-O asymmetric  stretching | 1126 cm^-1^ - C-O asymmetric stretching  1309 cm^-1^ - O-H in-plane bend |
| **Raman-specific bonds (cm^-1^)** | 1462 cm^-1^ - CH3 anti-symmetric vibration  2934 cm^-1^ - CH2 asymmetric stretching vibration modes  2975 cm^-1^ - CH3 asymmetric stretching vibration modes  1104 cm^-1^ - CCO skeleton stretching | 1454 cm^-1^ - CH3 anti-symmetric vibration  2923 cm^-1^ - CH2 asymmetric stretching vibration modes |
| **Similarities between FTIR and Raman peaks and assignments** | 881 cm^-1^ - C-C-O symmetric stretching vibration modes  1046 cm^-1^ - C–O stretching modes | 817 cm^-1^- Symmetric C-O stretch  950 cm^-1^ - CH3-C-CH3 stretching |

**References:**

1. Chan, A. P.; Chan, T. Y. Methanol as an unlisted ingredient in supposedly alcohol-based hand rub can pose serious health risk. *Int. J. Environ. Res. Public Health*. **2018**, *15*(7),1-16. DOI: 10.3390/ijerph15071440
2. Hickstein, D. D.; Goldfarbmuren, R.; Darrah, J.; Erickson, L.; Johnson, L. A. Rapid, accurate, and precise concentration measurements of a methanol–water mixture using Raman spectroscopy. *OSA Continuum*. **2018**, *1*(3), 1097-1110. DOI: 10.1364/OSAC.1.001097
3. Edwards, H. G. M.; Farwell, D. W.; Bowen, R. D. FT-Raman spectra of n-propanol and selected partially 2H-labelled analogues. *J. Mol. Struct*. **2007**, *832*(1-3), 184-190. DOI: 10.1016/j.molstruc.2006.08.018
4. Gu, Z.; Chen, W.; Du, L.; Zou, J.; Long, Z. Analysis of methyl ethyl ketone dissolved in transformer oil using laser Raman spectroscopy. In *2016 IEEE International Conference on High Voltage Engineering and Application (ICHVE)*, 2016; pp 1-4. DOI: 10.1109/ICHVE.2016.7800728.
5. Milanez, K. D. T. M.; Nóbrega, T. C. A.; Nascimento, D. S.; Insausti, M.; Band, B. S. F.; Pontes, M. J. C. Multivariate modeling for detecting adulteration of extra virgin olive oil with soybean oil using fluorescence and UV–Vis spectroscopies: A preliminary approach.*LWT-Food Sci. Technol*. **2017**; *85*, 9-15. DOI: 10.1016/j.lwt.2017.06.060
6. Wu, N.; Zhang, Y.; Ren, J.; Zeng, A.; Liu, J. Preparation of quercetin–nicotinamide cocrystals and their evaluation under in vivo and in vitro conditions. *RSC Adv*. **2020**, *10*(37), 21852-21859. DOI: 10.1039/D0RA03324C
7. Sallum, L. F.; Soares, F. L. F.; Ardila, J. A.; Carneiro, R. L. Optimization of SERS scattering by Ag-NPs-coated filter paper for quantification of nicotinamide in a cosmetic formulation. *Talanta*, **2014**, *118*, 353-358. DOI: 10.1016/j.talanta.2013.10.039
8. *Niacinamide*. https://webbook.nist.gov/cgi/cbook.cgi?ID=C98920&Mask=400. (accessed 2021-03-01)
9. Fan, B.; You, J.; Suo, Y.; Qian, C. A novel and sensitive method for determining vitamin B3 and B7 by pre-column derivatization and high-performance liquid chromatography method with fluorescence detection. *PloS one*, **2018**, *13*. DOI: 10.1371/journal.pone.0198102
10. León, A.; Reuquen, P.; Garín, C.; Segura, R.; Vargas, P.; Zapata, P.; Orihuela, P. A. FTIR and Raman characterization of TiO2 nanoparticles coated with polyethylene glycol as carrier for 2-methoxyestradiol. *Appl. Sci*. **2017**, *7*(1). DOI: 10.3390/app7010049
11. Mohamed, E. M. A.; Eisa, W. H.; Abdel-Baset, T. A.; Mahrous, S. Preparation and characterization of PEG-assisted growth of colloidal Ag nanoparticles. *IJASR*. **2017**, *3*(6), 65–68. DOI: 10.7439/ijasr
12. *Propylene glycol*. https://pubchem.ncbi.nlm.nih.gov/compound/Propylene-glycol#section=FTIR-Spectra. (accessed 2021-03-01)
13. De Veij, M.; Vandenabeele, P.; De Beer, T.; Remon, J. P.; Moens, L. Reference database of Raman spectra of pharmaceutical excipients. *J. Raman Spectrosc*. **2009**, *40*(3), 297-307. DOI: 10.1002/jrs.2125
14. Damasceno, J. P. L.; dos Santos Giuberti, C.; Gonçalves, R. D. C. R.; Kitagawa, R. R. Preformulation study and influence of DMSO and propylene glycol on the antioxidant action of isocoumarin paepalantine isolated from Paepalanthus bromelioides. *Rev. Bras. Farmacogn*. **2015**, *25*(4), 395-400. DOI: 10.1016/j.bjp.2015.07.008
15. Magalhães, A. S. G.; Almeida Neto, M. P.; Bezerra, M. N.; Ricardo, N. M.; Feitosa, J. Application of FTIR in the determination of acrylate content in poly (sodium acrylate-co-acrylamide) superabsorbent hydrogels. *Quím. Nova*. **2012**, *35*(7), 1464-1467. DOI: 10.1590/S0100-40422012000700030
16. Jöhnck, M.; Müller, L.; Neyer, A.; Hofstraat, J. W. Quantitative determination of unsaturation in photocured halogenated acrylates and methacrylates by FT-IR and Raman-spectroscopy and by thermal analysis. *Polymer*. **1999**, *40*(13), 3631-3639. DOI: 10.1016/S0032-3861(98)00596-5
17. Todica, M.; Pop, C. V.; Stefan, R.; Nagy, M.; Garabagiu, S. Spectroscopic investigation of some poly (acrylic acid) gels with embedded gold nanoparticles*. Stu. U. Babes-Bol. Chem*. **2015**, *60*(1), 19-28.
18. *N,N,N',N'-Tetrakis(2-hydroxypropyl)ethylenediamine.* https://pubchem.ncbi.nlm.nih.gov/compound/Edetol. (accessed 2021-03-01)
19. *1,2-Benzenedicarboxylic acid*. https://spectrabase.com/spectrum/CLExDedHNPk. (accessed 2021-03-01)
20. Thomas O, Burgess C. *UV-visible spectrophotometry of water and wastewater*. Elsevier, 2017.
21. Ahmed, M. H., Byrne, J. A.; McLaughlin, J. A. D.; Elhissi, A.; Ahmed, W. Comparison between FTIR and XPS characterization of amino acid glycine adsorption onto diamond-like carbon (DLC) and silicon doped DLC.  *Appl. Surf. Sci*. **2013**, *273*, 507-514. DOI: 10.1016/j.apsusc.2013.02.070
22. Numata, Y.; Otsuka, M.; Yamagishi, K.; Tanaka, H. Quantitative determination of glycine, alanine, aspartic acid, glutamic acid, phenylalanine, and tryptophan by Raman spectroscopy. *Anal. Lett*. **2017**, *50*(4), 651-662. DOI: 10.1080/00032719.2016.1193189
23. Dimitrijević, S.; Rajčić-Vujasinović, M.; Alagić, S.; Grekulović, V.; Trujić, V. Formulation and characterization of electrolyte for decorative gold plating based on mercaptotriazole. *Electrochim. Acta*. **2013**, *104*, 330-336. DOI: 10.1016/j.electacta.2013.04.123
24. Perucho, J.; Gonzalo-Gobernado, R.; Bazan, E.; Casarejos, M. J.; Jiménez-Escrig, A.; Asensio, M. J.; Herranz, A. S. Optimal excitation and emission wavelengths to analyze amino acids and optimize neurotransmitters quantification using precolumn OPA-derivatization by HPLC. *Amino Acids*, **2015**, *47*(5), 963-973. DOI: 10.1007/s00726-015-1925-1
25. *Triethanolamine*. https://pubchem.ncbi.nlm.nih.gov/compound/Triethanolamine#section=Other-MS. (accessed 2021-03-01)
26. Conterosito E, Croce G, Palin L, Boccaleri E, van Beek W, Milanesio M. Crystal structure and solid-state transformations of Zn–triethanolamine–acetate complexes to ZnO. *CrystEngComm*. **2012**, *14*(13), 4472-4477. DOI: 10.1039/C2CE06468E
27. *Triethanolamine*. https://webbook.nist.gov/cgi/cbook.cgi?ID=C121448&Mask=400. (accessed 2021-03-01)
28. Pramod, K.; Suneesh, C. V.; Shanavas, S.; Ansari, S. H.; Ali, J. Unveiling the compatibility of eugenol with formulation excipients by systematic drug-excipient compatibility studies. *J. Anal. Sci. Tech*. **2015**, *6*(34), 1-14. DOI: 10.1186/s40543-015-0073-2
29. Sahoo, S.; Chakraborti, C. K.; Behera, P. K. FTIR and Raman Spectroscopic Investigations of Ofloxacin / Carbopol940 Mucoadhesive Suspension. *Int. J. Pharmtech Res*. **2012**, *4*(1), 382-392. DOI:
30. *2-Phenoxyethanol*. https://spectrabase.com/spectrum/LR2ehDaeFgt. (accessed 2021-03-01)
31. *2-Phenoxyethanol*. https://spectrabase.com/spectrum/DrWOkoD52xr. (accessed 2021-03-01)
32. *2-Phenoxyethanol*. https://spectrabase.com/spectrum/5ql3YCUovwb. (accessed 2021-03-01)
33. Fathi, M.; Nasrabadi, M. N.; Varshosaz, J. Characteristics of vitamin E-loaded nanofibres from dextran. *Int. J. Food Prop*. **2017**, *20*(11), 2665-2674. DOI: 10.1080/10942912.2016.1247365
34. Beattie, J. R.; Maguire, C.; Gilchrist, S.; Barrett, L. J.; Cross, C. E.; Possmayer, F.; Ennis, M.; Elborn, J. S.; Curry, W. J.; McGarvey, J. J.; Schock, B. C. The use of Raman microscopy to determine and localize vitamin E in biological samples. *The FASEB Journal*, **2007**; *21*(3), 766-776. DOI: 10.1096/fj.06-7028com
35. Demirkaya-Miloglu, F.; Kadioglu, Y.; Senol, O.; Yaman, M. E. Spectrofluorimetric determination of α-tocopherol in capsules and human plasma. *Indian J. Pharma. Sci*. **2013**, *75*(5), 563-568.
36. Lim, Z. X.; Cheong, K. Y. Effects of drying temperature and ethanol concentration on bipolar switching characteristics of natural Aloe vera-based memory devices. *Phys. Chem. Chem. Phys*. **2015**, *17*(40), 26833-26853. DOI: 10.1039/C5CP04622J
37. Carac, A.; Boscencu, R.; Patriche, S.; Dinica, R. M.; Carac, G.; Gird, C. E. Antioxidant and Antimicrobial Potential of Extracts from Aloe vera Leaves. *Rev Chim (Burcharest)*, **2016**, *4*(67), 654-658.
38. Jain, S.; Mehata, M. S. Medicinal plant leaf extract and pure flavonoid mediated green synthesis of silver nanoparticles and their enhanced antibacterial property. *Sci. Rep*. **2017**, *7*(1), 1-13. DOI: 10.1038/s41598-017-15724-8
39. Basak, P.; Paul, P.; Kundu, T.; Mallick, P. Evaluating the anti-microbial effect of eugenol extracted from ocimum sanctum. *J. Drug Deliv. Ther*. **2016**, *6*(5), 1-5. DOI: 10.22270/jddt.v6i5.1307
40. Choudhury, R.; Majumder, M.; Roy, D. N.; Basumallick, S.; Misra, T. K. Phytotoxicity of Ag nanoparticles prepared by biogenic and chemical methods. *Int. Nano Lett*. **2016**, *6*(3), 153-159. DOI: 10.1007/s40089-016-0181-z
